# Identification of anthocyanins in deep colored berries and grains in China

**DOI:** 10.1016/j.fochx.2024.101602

**Published:** 2024-06-27

**Authors:** Boyu Xie, Miaoshu Wang, Dong Yang

**Affiliations:** a College of Food Science & Nutritional Engineering, China Agricultural University, Beijing 100083, China; b Beijing Key Laboratory of Functional Food from Plant Resources, College of Food Science & Nutritional Engineering, China Agricultural University, Beijing 100083, China

**Keywords:** Anthocyanin, Identification, Authentic standards, Berries, Blue honeysuckle, Black grains

## Abstract

Identification of anthocyanin relies heavily on authentic anthocyanin standards and the detection instruments employed, and both of these made tremendous improvement in the past decades. Here, with 118 authentic anthocyanin standards and state-of-the-art detection method, we comprehensively analyze the anthocyanin composition of the most commonly seen, deep-colored berries and grains in China. Cyanidin-3-*O*-glucoside is the overwhelmingly major anthocyanin in all the berries and grains examined, including blue honeysuckle, blueberry, cranberry, elderberry, mulberry, black rice, and black soybean, which accounts for an average of 82% of the total anthocyanins with a little variation between them. Peonidin-3-*O*-glucoside is the second most abundant anthocyanin ranging from 2.6%–14.9% of the total anthocyanins. Pelargonidin-3,5-*O*-diglucoside is only found in blue honeysuckle, and besides that, berries and grains share a dominant portion of common anthocyanins among them. This study primes the survey of anthocyanin in common Chinese foods for the establishment of a nutrition database.

## Highlights

• The anthocyanin in major Chinese berries and grains were identified.
• Total of 118 authentic anthocyanin standards were used.
• Cyanidin-3-*O*-glucoside is the overwhelmingly major anthocyanin.
• Berries and grains share a dominant portion of common anthocyanins.

## 1 Introduction

Anthocyanins are chemicals composed of an aglycone (the anthocyanidin), sugar(s) and usually acyl group(s) which exhibit multiple health benefit to humans (Liang, Liang, Guo, & Yang, 2021). Anthocyanins are structurally diverse due to not only different hydroxyl and/or methoxyl substitutions at the R1, R2, R3 positions of the flavylium (2-phenylbenzopyrilium) structure, but also different sugar groups attached(Liang et al., 2021). Subsequently, their diverse structures bring about different functions. For example, cyanidin-glucoside could inhibit Aβ aggregation and may help to enhance the cognitive function of mice via synergistically functioning with other anthocyanins(N. Y. Kim & Lee, 2016; Wong, Musgrave, Harvey, & Smid, 2013). On the other hand, cyanidin-sophoroside which carries different sugar group, exhibits antioxidative activity(Proteggente et al., 2002). Peonidin-glucoside, which differs from cyanidin-glucoside from the substitution at the R1 position, improves plasma antioxidant capacity, inhibits nitric oxide production in macrophages and suppresses reactive oxygen species(Hu, Zawistowski, Ling, & Kitts, 2003; Ramirez-Tortosa et al., 2001; Wang & Mazza, 2002). Thus, it is meaningful to fully utilize anthocyanins from food and agricultural byproducts(Zhou & Yang, 2022). Currently, it is difficult to obtain commercial products with anthocyanins as the main functioning components and dietary is still the main source of anthocyanin intake worldwide (Zhang et al., 2021).

Currently, consumers are intuitively informed that colored foods are often beneficial to their health, and some of them know that it is partially because of the anthocyanins rich in these foods. However, little is known about the variety and content of anthocyanins a specific food contains. This jeopardizes the intake of specific anthocyanin for the purpose of precision nutrition. For this, the Nutrient Data Laboratory of the United States Department of Agriculture established a flavonoid database (FDB) in 2003 and a proanthocyanidin database (PDB) in 2004(Harnly et al., 2006). Survey of anthocyanin contents in common foods was executed with valid national sampling plan and analytical methods in the United States (Wu et al., 2006; Wu & Prior, 2005). Occasionally, the anthocyanin content of a local anthocyanin-rich food is also reported for reference elsewhere in the world. For example, the anthocyanin contents of wild blueberries in Québec area were reported previously(Nicoue, Savard, & Belkacemi, 2007). However, there is currently no data base of anthocyanin distribution in the common foods in China.

Thus, here we determine the anthocyanin compositions of the most colored berries seen in Chinese markets, including blueberry, blue honeysuckle (*Lonicera caerulea* L.), cranberry, elderberry, mulberry, and the most colored grains commonly available to Chinese citizens, including black rice and black soybean. All of these berries and grains are reported exhibiting antioxidative activity associated with their anthocyanin contents(Domínguez et al., 2020; Felgus-Lavefve, Howard, Adams, & Baum, 2022; I. Kim & Lee, 2020; Kumar et al., 2023; Wang et al., 2017; Zhang et al., 2023; Zhang, Zhang, Zhang, & Liu, 2010). This study aims to comprehensively analyze almost all possible anthocyanin components in the commonly consumed, deep colored berries and grains, to lay a foundation for the precise intaking of dietary anthocyanins.

## 2 Materials and methods

### 2.1 Materials

Berries (including cranberry, elderberry, and mulberry) and grains (including black rice and black soybean) were purchased from Shaanxi Evergreen Biotech. Ltd. (Xi’an, Shaanxi, China). And these above berries and grains were collected in Shaanxi Province in 2023. Blueberry was collected from the Fenglin County, Heilongjiang Province in May 2023. Blue honeysuckle (cultivar of Lanjingling) collected in June 2023 was kindly gifted from Prof. Junwei Huo at College of Horticulture and Landscape Architecture, Northeast Agricultural University, Harbin, Heilongjiang Province.

Chemicals including absolute ethanol and acetic acid were purchased from Modern Oriental Fine Chemistry (Beijing, China), formic acid was purchased from Sigma-Aldrich (St. Louis, USA), hydrochloric acid was purchased from Sinopharm Chemical Reagent Co., ltd (Beijing, China), methanol was purchased from Merck (NJ, USA), potassium chloride and sodium acetate were purchased from Xilong Scientific (Shantou, Guangdong Province, China).

### 2.2 Anthocyanin extraction

Anthocyanins were extracted from the above berries and grains principally the same as described previously with minor modifications(Zhou & Yang, 2022). Briefly, berries and grains were mixed with 79% ethanol of pH 7.0 at a material liquid ratio of 1:15, smashed, and incubated at 64 °C for 50 min with the application of a VGT-1730QTD ultrasonic water bath (GT Sonic, Meizhou, Guangdong Province, China). The extract solution was centrifuged at 4000 rpm for 15 min at room temperature to remove precipitates, and filtered with a 0.45 μM PTFE microfiltration membrane.

### 2.3 Anthocyanin analysis

The above extracted anthocyanins were 10-folds diluted into a solution containing 50% methanol and 0.1% hydrochloric acid, vortexed for 5 min, sonicated for 5 min and centrifuged at 12000 r/min for 3 min. The supernatant was filtered through a 0.22 μM microfiltration membrane right before Ultra Performance Liquid Chromatography (ExionLC™, SCIEX) and Tandem Mass Spectrometry (QTRAP 6500+) analysis (UPLC-MS/MS).

On a ACQUITY UPLC BEH C18 column (1.7 μm, 2.1 mm × 100 mm) equilibrated with a buffer containing 0.1% formic acid, 2 μL anthocyanin solution was loaded and eluted with increasing concentration of another methanol solution containing 0.1% formic acid. The flow rate was set at 0.35 mL/min and column temperature was set at 40 °C. The eluted anthocyanins were electrospray ionized and detected by their declustering potential and collision energy.

A total of 118 different authentic anthocyanin/proanthocyanin standards were applied to the above detection method. Among them, 46 were detected and a standard curve was established for each (Table 1) with concentrations of 0.01 ng/mL, 0.05 ng/mL, 0.1 ng/mL, 0.5 ng/mL, 1 ng/mL, 5 ng/mL, 10 ng/mL, 50 ng/mL, 100 ng/mL, 500 ng/mL, 1000 ng/mL, 2000 ng/mL, 5000 ng/mL loaded on the UPLC-MS/MS to establish the standard curve. Sample concentration was the input variable and corresponding peak area was the output.

**Table 1:** Standard curves of authentic anthocyanins.

**Sample**	**Retention Time (min)**	**Equation**		**R**^**2**^**value**
		**Slope (10**^**4**^**)**	**intercept**	
Cyanidin-3-*O*-(6-*O*-malonyl-β-D-glucoside)	10.36	5.85106	1941.83968	0.99922
Cyanidin-3-(malonyl)glucoside-5-rhamnoside	11.24	6.93442	16,914.79855	0.99982
Cyanidin-3,5-*O*-diglucoside	5.52	6.93442	16,914.79855	0.99982
Cyanidin-3-[6″-(acetyl)xylosyl]-xyloside	8.73	6.93442	16,914.79855	0.99982
Cyanidin-3-gentiobioside	6.41	6.93442	16,914.79855	0.99982
Cyanidin-3-*O*-(6″-*O*-acetyl)glucoside	10.86	6.93442	16,914.79855	0.99982
Cyanidin-3-*O*-(6″-*O*-caffeoyl)rhamnoside	11.79	6.93442	16,914.79855	0.99982
Cyanidin-3-*O*-(6″-*O*-coumaryl-galloy)glucoside	9.17	6.93442	16,914.79855	0.99982
Cyanidin-3-*O*-(6″-*O*-sinapoyl)glucoside-5-(6‴-*O*-xylosyl)glucoside	7.51	6.93442	16,914.79855	0.99982
Cyanidin-3-*O*-(malonyl)(glucoside)galactoside	8.23	6.93442	16,914.79855	0.99982
Cyanidin-3-*O*-(tartaryl)rhamnoside-5-*O*-glucoside	8.21	6.93442	16,914.79855	0.99982
Cyanidin-3-*O*-arabinoside	7.97	9.75946	33,324.9	0.99514
Cyanidin-3-*O*-galactoside	6.98	5.08707	19,858.06510	0.99808
Cyanidin-3-*O*-glucoside	7.54	2.01520	12,732.03820	0.99994
Cyanidin-3-*O*-rutinoside-glucoside	8.91	6.93442	16,914.79855	0.99982
Cyanidin-3-*O*-sambubioside	7.67	10.4999	1497.48836	0.99992
Cyanidin-3-*O*-sophoroside	6.96	6.13307	4030.89432	0.99647
Cyanidin-3-*O*-xyloside	9.77	6.98974	12,839.36927	0.99866
Cyanidin-3-xylosyl-galactoside	6.32	6.93442	16,914.79855	0.99982
Delphinidin-3-*O*-galactoside	6.05	8.01900	232,198	0.99769
Delphinidin-3-*O*-glucoside	6.5	9.03420	95,521.7	0.99806
Delphinidin-3-*O*-rutinoside	7.13	5.49410	27,776.94341	0.99905
Delphinidin-3-*O*-rutinoside-5-*O*-glucoside	7.14	4.06457	21,429.00049	0.9997
Delphinidin-3-*O*-sambubioside	6.42	7.43101	2920.08858	0.99601
Delphinidin-3-*O*-sophoroside	6	4.06457	21,429.00049	0.9997
Malvidin-3-*O*-arabinoside	10.03	5.10190	51,480.3	0.99961
Malvidin-3-*O*-galactoside	9.16	7.63197	81,676.9	0.9987
Malvidin-3-*O*-glucoside	9.56	7.6095	82,798.0	0.99675
Naringenin	13.07	2.22462	5596.40889	0.99341
Pelargonidin-3,5-*O*-diglucoside	6.35	4.69933	19,725.9	0.9999
Pelargonidin-3-*O*-galactoside	7.88	4.69933	19,725.9	0.9999
Pelargonidin-3-*O*-glucoside	8.51	8.05631	22,773.82442	0.99642
Pelargonidin-3-*O*-sophoroside	7.81	4.69933	19,725.97265	0.9999
Peonidin-3,5-*O*-diglucoside	6.86	6.92159	38,305.3	0.99924
Peonidin-3-*O*-arabinoside	9.58	7.36793	61,327.1	0.99731
Peonidin-3-*O*-glucoside	9.16	3.55009	3198.18386	0.99936
Petunidin-3-*O*-glucoside	8.21	8.54467	49,884.3	0.99823
Petunidin-3-*O*-rutinoside	8.79	8.54467	49,884.3	0.99823
Procyanidin A1	7.32	3.61842	9966.80621	0.99945
Procyanidin A2	9.25	0.996608984	6218.93720	0.9978
Procyanidin B1	3.91	0.517029929	830.76938	0.99948
Procyanidin B2	5.54	0.529383641	4429.7931	0.99941
Procyanidin B3	3.54	0.481134518-	4115.88474	0.99949
Procyanidin B4	4.57	0.616441078	1129.85159	0.99961
Procyanidin C1	6.81	0.089788359	3315.42598	0.99944
Quercetin-3-*O*-glucoside	11.28	0.957826771	4231.80933	0.99622

## 3 Results and discussion

### 3.1 Establishment of an exhaustive detection method

The UPLC-MS/MS detection method is currently the most widely used one to detect anthocyanins and its detection capacity mainly depends on the standard chemicals used. In our previous identification of anthocyanins from the grape pomace, only 15 authentic standards were used and thus the maximum number of anthocyanin species we could detect was limited(Zhou & Yang, 2022). Among them, the sugars are limited to glucoside, acetylglucoside, and coumaroylglucoside, and the anthocyanidins are limited to cyanidin, peonidin, delphinidin, petunidin, and malvidin.

Here, we employed 118 authentic anthocyanin standards among which 46 were detected (Table 1). For anthocyanidins, naringenin, pelargonidin, and quercetin were additionally employed. For sugars, arabinoside, diglucoside, galactoside, gentiobioside, rhamnoside, rutinoside, sambubioside, sophoroside, and xyloside were additionally used. Furthermore, procyanidin A1, A2, B1, B2, B3, B4, and C1 were also studied. In the 0.01 ng/mL to 5000 ng/mL range, all the authentic anthocyanins exhibited good linear response, with all the R^2^ values higher than 0.99, to the multiple reaction monitoring (MRM) detection mode (Table 1). This enables us to accurately determine the composition and proportion of each anthocyanin in the sample.

**Fig. 1:**
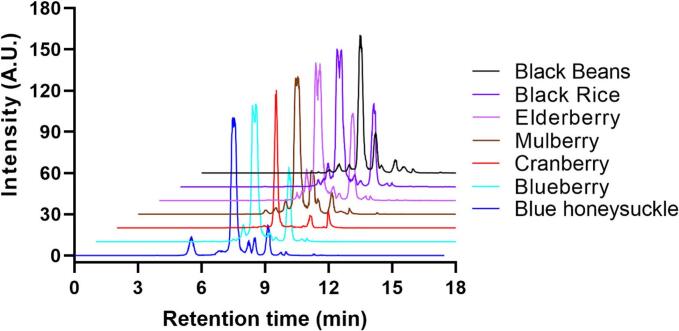
Particle flow diagram of anthocyanin components in common berries and grains. The Multiple Reaction Monitoring mode of MS/MS detection exhibited multiple peaks, major and minor, indicating a mixture of anthocyanins found in each of the berry and grain extraction.

As shown in Fig. 1, there are numerous peaks in the particle flow diagram generated by the MRM mode with the MS/MS instrument, indicating a mixture of different anthocyanins in each of the berry and grain samples analyzed. Since our detection mode allows quantitative analysis of anthocyanins, it is obvious that in each sample there is a couple of major anthocyanin components and several minor anthocyanin components. Each component and its proportion is identified and discussed in the following.

### 3.2 Major and minor anthocyanin components in berries and grains

Multiple peaks are detected for all of the 7 samples studied here, and apparently some anthocyanins are overwhelmingly richer than others. The identification and proportion of anthocyanins are quantitatively analyzed, and found that the distribution of anthocyanin in all the samples are highly heterogenous. To better analyze and describe such unevenly distributed anthocyanins, we define that anthocyanins accounting for more than 1% of the total anthocyanins are major components, and these accounting for less than 1% of the total anthocyanins are minor ones.

**Fig. 2:**
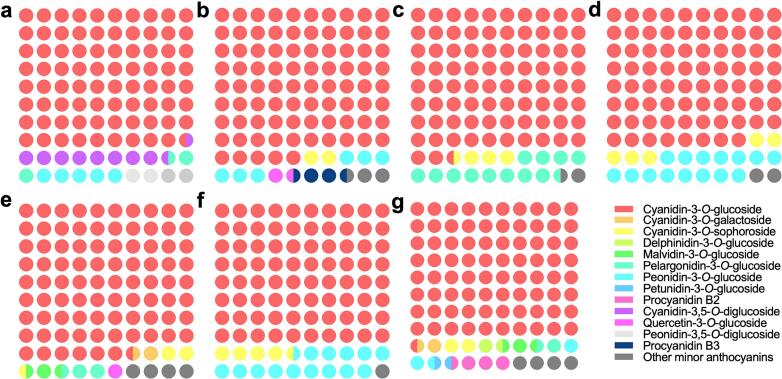
The major anthocyanin components of berries and grains detected. Anthocyanins accounting for more than 1% of the total anthocyanins are defined as major anthocyanins, and their distribution in different berries and grains are shown here as (a) blue honeysuckle, (b) cranberry, (c) blueberry, (d) elderberry, (e) mulberry, (f) black rice, and (g) black soybean. (For interpretation of the references to colour in this figure legend, the reader is referred to the web version of this article.)

Here, it is seen in all the berries and grains, cyanidin-3-*O*-glucoside is the main anthocyanin which accounts for an average of 81.9% of the total anthocyanins and its content slightly deviates among different samples (Fig. 2). In blue honeysuckle, the 2nd most abundant anthocyanin is cyanidin-3,5-*O*-diglucoside, which accounts for 8.7% of the total anthocyanin and the 3rd most abundant one is peonidin-3-*O*-glucoside, which accounts for 4.9% of the total anthocyanin (Fig. 2a). In cranberry, the following anthocyanins are peonidin-3-*O*-glucoside and procyanidin B3 (Fig. 2b). In blueberry, peonidin-3-*O*-glucoside is accounting for 12.6% of the total anthocyanins (Fig. 2c). In elderberry, peonidin-3-*O*-glucoside is accounting for 14.9% of the total anthocyanins (Fig. 2d). In mulberry, there is no peonidin-3-*O*-glucoside detected and no other anthocyanins are accounting more than 3% of the total anthocyanins (Fig. 2e). In black rice, peonidin-3-*O*-glucoside is accounting for 14.4% of the total anthocyanins and cyanidin-3-*O*-sophoroside is accounting for 4.2% (Fig. 2f). In black soybean, peonidin-3-*O*-glucoside is accounting for 2.6% of the total anthocyanins (Fig. 2g) and other major anthocyanins are around the same proportion. In almost all the samples examined, cyanidin-3-*O*-glucoside is the overwhelmingly main anthocyanin, followed by peonidin-3-*O*-glucoside except in mulberry.

**Fig. 3:**
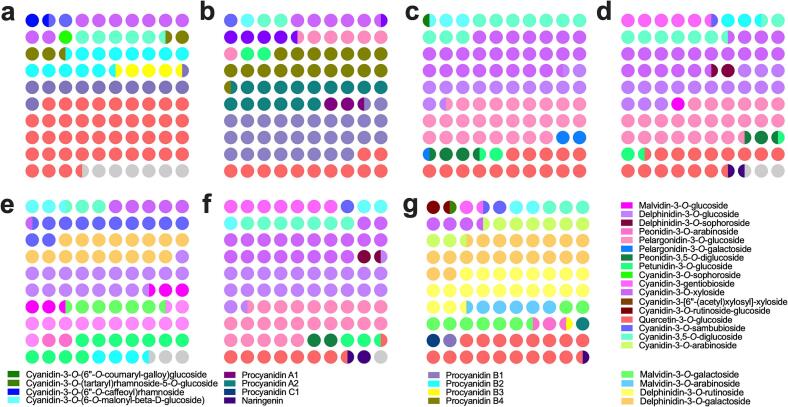
The minor anthocyanin components of berries and grains detected. Anthocyanins accounting for less than 1% of the total anthocyanins are defined as minor anthocyanins, and their distribution in different berries and grains are shown here as (a) blue honeysuckle, (b) cranberry, (c) blueberry, (d) elderberry, (e) mulberry, (f) black rice, and (g) black soybean. (For interpretation of the references to colour in this figure legend, the reader is referred to the web version of this article.)

As for the minor anthocyanins, cyanidin-3-*O*-xyloside is present in all the samples examined here (Fig. 3). Cyanidin-3-*O*-(6-*O*-malonyl-β-D-glucoside), cyanidin-3,5-*O*-diglucoside and petunidin-3-*O*-glucoside are present among all the samples except blue honeysuckle. Quercetin-3-*O*-glucoside is present among all the samples except in mulberry. Pelargonidin-3,5-*O*-diglucoside is only found in blue honeysuckle, accounting for 985.6 ppm of the total anthocyanins, and not found in any other samples (Fig. 3a). There are no other specific anthocyanins exclusively present in other samples (Fig. 3b-g). Interestingly, there are more anthocyanins evenly distributed in the minor anthocyanin group of black soybeans than in other samples (Fig. 3g).

The anthocyanin contents of blue honeysuckle were determined two decades ago with 6 authentic standards, including cyanidin 3,5-diglucoside, cyanidin 3-glucoside, cyanidin 3-rutinoside, pelargonidin 3-glucoside, peonidin 3-glucoside, peonidin 3-rutinoside(Chaovanalikit, Thompson, & Wrolstad, 2004). Here with more authentic anthocyanin standards and more accurate MS/MS detection mode, 19 additional anthocyanins were determined, including cyanidin-3-*O*-(6″-*O*-caffeoyl)rhamnoside, cyanidin-3-[6″-(acetyl)xylosyl]-xyloside, cyanidin-3-*O*-sambubioside, cyanidin-3-*O*-(6-*O*-malonyl-β-D-glucoside), cyanidin-3-*O*-xyloside, cyanidin-3-*O*-sophoroside, cyanidin-3-*O*-arabinoside, delphinidin-3-*O*-rutinoside-5-*O*-glucoside, delphinidin-3-*O*-glucoside, delphinidin-3-*O*-rutinoside, pelargonidin-3,5-*O*-diglucoside, peonidin-3,5-*O*-diglucoside, procyanidin A2, B1, B2, B3, B4, quercetin-3-*O*-glucoside and naringenin.

### 3.3 Common anthocyanin components in berries and grains

**Fig. 4:**
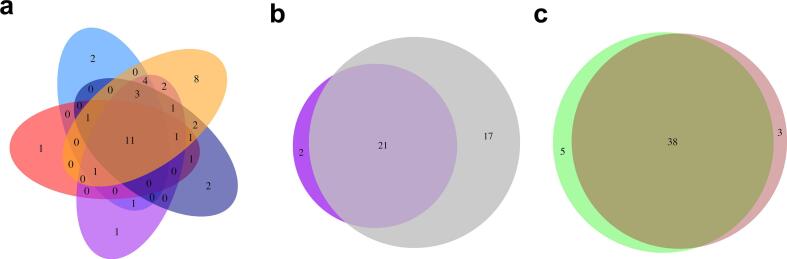
Venn diagram of common anthocyanins. (a) Venn diagram of anthocyanins between five berry samples examined. Blue indicates blueberry, orange indicates mulberry, dark blue indicates blue honeysuckle, purple indicates elderberry, and red indicates cranberry. (b) Venn diagram of anthocyanins between black rice and black soybean. Purple indicates black rice and grey indicates black soybean. (c) Venn diagram of anthocyanins between berries and grains. Green indicates berries and brown indicates grains. (For interpretation of the references to colour in this figure legend, the reader is referred to the web version of this article.)

The common anthocyanins in berries and grains are analyzed with Venn diagram. All the 5 berries share a core of 11 common anthocyanins, including cyanidin-3-*O*-glucoside, cyanidin-3-*O*-sambubioside, cyanidin-3,5-*O*-diglucoside, cyanidin-3-*O*-(6-*O*-malonyl-β-D-glucoside), cyanidin-3-*O*-sophoroside, cyanidin-3-*O*-xyloside, pelargonidin-3-*O*-glucoside, delphinidin-3-*O*-glucoside, procyanidin B3, peonidin-3-*O*-glucoside and quercetin-3-*O*-glucoside (Fig. 4a). There are also specific anthocyanins in each berry only. In blueberry, 2 anthocyanins pelargonidin-3-*O*-galactoside and cyanidin-3-*O*-(6″-*O*-acetyl)glucoside are specifically present. Cyanidin-3-(malonyl)glucoside-5-rhamnoside is only present in elderberry while procyanidin A1 is only present in cranberry. Cyanidin-3-*O*-(6″-*O*-caffeoyl)rhamnoside and pelargonidin-3,5-*O*-diglucoside are only present in blue honeysuckle. In mulberry, there are 8 anthocyanins present only in this berry, including cyanidin-3-*O*-(tartaryl)rhamnoside-5-*O*-glucoside, cyanidin-3-*O*-galactoside, cyanidin-3-*O*-(malonyl)(glucoside)galactoside, delphinidin-3-*O*-sambubioside, delphinidin-3-*O*-galactoside, malvidin-3-*O*-arabinoside, malvidin-3-*O*-galactoside and pelargonidin-3-*O*-sophoroside. Most of the berries harbors very few specific anthocyanins except mulberry.

Similarly, black rice and black soybean share a core of 21 common anthocyanins, including cyanidin-3-*O*-rutinoside-glucoside, cyanidin-3-*O*-(6″-*O*-coumaryl-galloy)glucoside, cyanidin-3-*O*-(6″-*O*-caffeoyl)rhamnoside, cyanidin-3-[6″-(acetyl)xylosyl]-xyloside, cyanidin-3-gentiobioside, cyanidin-3-*O*-sambubioside, cyanidin-3-*O*-(6-*O*-malonyl-β-D-glucoside), cyanidin-3,5-*O*-diglucoside, cyanidin-3-*O*-xyloside, cyanidin-3-*O*-sophoroside, cyanidin-3-*O*-arabinoside, delphinidin-3-*O*-glucoside, delphinidin-3-*O*-rutinoside, malvidin-3-*O*-glucoside, pelargonidin-3-*O*-glucoside, peonidin-3-*O*-glucoside, peonidin-3,5-*O*-diglucoside, peonidin-3-*O*-arabinoside, petunidin-3-*O*-glucoside, quercetin-3-*O*-glucoside and naringenin (Fig. 4b). Delphinidin-3-*O*-sophoroside and cyanidin-3-*O*-(6″-*O*-sinapoyl)glucoside-5-(6‴-*O*-xylosyl)glucoside are specifically present in black rice. While cyanidin-3-*O*-(tartaryl)rhamnoside-5-*O*-glucoside, cyanidin-3-*O*-galactoside, cyanidin-3-*O*-(malonyl)(glucoside)galactoside, cyanidin-3-xylosyl-galactoside, delphinidin-3-*O*-rutinoside-5-*O*-glucoside, delphinidin-3-*O*-sambubioside, delphinidin-3-*O*-galactoside, malvidin-3-*O*-arabinoside, malvidin-3-*O*-galactoside, pelargonidin-3-*O*-sophoroside, petunidin-3-*O*-rutinoside and proanthocyanidin A2, B1, B2, B3, B4, C1 were specifically present in black soybean.

The anthocyanins between berries and grains are compared. Five anthocyanins are present only in berry samples, including cyanidin-3-*O*-(6″-*O*-caffeoyl)rhamnoside, cyanidin-3-*O*-(6″-*O*-acetyl)glucoside, cyanidin-3-(malonyl)glucoside-5-rhamnoside, pelargonidin-3-*O*-galactoside and procyanidin A1. And there are 3 anthocyanins present only in grain samples, including cyanidin-3-xylosyl-galactoside, petunidin-3-*O*-rutinoside and procyanidin C1 (Fig. 4c).

Among all the 118 authentic anthocyanin standards used, only 46 are found in all berry samples or grains samples. These 72 anthocyanins not found in any of these samples are: petunidin-3,5-*O*-diglucoside, petunidin-3-*O*-(6-*O*-malonyl-β-D-glucoside), petunidin-3-*O*-(6-*O*-p-coumaroyl)-glucoside, petunidin-3-*O*-5-*O*-(6-*O*-coumaroyl)-diglucoside, petunidin-3-*O*-arabinoside, petunidin-3-*O*-galactoside, petunidin-3-*O*-sambubioside, petunidin-3-*O*-sambubioside-5-*O*-glucoside, petunidin-3-*O*-sophoroside, delphinidin-3,5-*O*-diglucoside, delphinidin-3-O-(6-*O*-acetyl)-glucoside, delphinidin-3-*O*-(6-*O*-malonyl)-glucoside-3′-glucoside, delphinidin-3-*O*-(6-*O*-malonyl-β-D-glucoside), delphinidin-3-*O*-(6-*O*-p-coumaroyl)-glucoside, delphinidin-3-*O*-5-*O*-(6-*O*-coumaroyl)-diglucoside, delphinidin-3-*O*-arabinoside, delphinidin-3-*O*-rhamnoside, delphinidin-3-*O*-sambubioside-5-*O*-glucoside, afzelin, chalcone, dihydrokaempferol, dihydromyricetin, kaempferol-3-*O*-rutinoside, naringenin-7-*O*-glucoside, quercetin-3-*O*-glucoside, rutin, malvidin-3,5-*O*-diglucoside, malvidin-3-*O*-(6″-acetylglucoside)-5-glucoside, malvidin-3-*O*-(6-*O*-malonyl-β-D-glucoside), malvidin-3-*O*-(6-*O*-p-coumaroyl)-glucoside, malvidin-3-*O*-5-*O*-(6-O-coumaroyl)-diglucoside, malvidin-3-*O*-rutinoside, malvidin-3-*O*-sambubioside, malvidin-3-*O*-sambubioside-5-*O*-glucoside, malvidin-3-*O*-sophoroside, peonidin-3-(caffeoyl-glucosyl-glucoside)-5-glucoside, peonidin-3-*O*-(6″-ferulylsophoroside)-5-glucoside, peonidin-3-*O*-(6-*O*-malonyl-β-D-glucoside), peonidin-3-*O*-(6-*O*-p-coumaroyl)-glucoside, peonidin-3-*O*-5-*O*-(6-*O*-coumaroyl)-diglucoside, peonidin-3-*O*-caffeoyl-feruloyl-sophoroside-5-glucoside, peonidin-3-*O*-galactoside, peonidin-3-*O*-P-hydroxybenzoylsophoroside-5-glucoside, peonidin-3-*O*-rutinoside, peonidin-3-*O*-sambubioside, peonidin-3-O-sambubioside-5-*O*-glucoside, peonidin-3-*O*-sophoroside, peonidin-3-sophoroside-5-glucoside, cyanidin-3-(6″-caffeylsophoroside)-5-glucoside, cyanidin-3-(6-*O*-p-caffeoyl)-glucoside, cyanidin-3,5,3′-*O*-triglucoside, cyanidin-3,5-*O*-diglucoside, cyanidin-3-*O*-(6″-ferulylsophoroside)-5-glucoside, cyanidin-3-*O*-(6-*O*-p-coumaroyl)-glucoside, cyanidin-3-*O*-5-*O*-(6-*O*-coumaroyl)-diglucoside, cyanidin-3-*O*-rutinoside-5-*O*-glucoside, cyanidin-3-*O*-sambubioside-5-*O*-glucoside, pelargonidin, pelargonidin-3-(6″-caffeylsophoroside)-5-glucoside, pelargonidin-3-*O*-(6″-ferulylsambubioside)-5-*O*-(malonyl)-glucoside, pelargonidin-3-*O*-(6-*O*-malonyl-β-D-glucoside), pelargonidin-3-*O*-(6-*O*-p-coumaroyl)-glucoside, pelargonidin-3-*O*-[2-*O*-glucosyl-6-*O*-p-coumaroyl-glucoside]-5-*O*-glucoside, pelargonidin-3-*O*-[6-*O*-feruloyl-2-*O*-glucosyl-glucoside]-5-*O*-glucoside, pelargonidin-3-*O*-5-*O*-(6-*O*-coumaroyl)-diglucoside, pelargonidin-3-*O*-arabinoside, pelargonidin-3-*O*-rutinoside, pelargonidin-3-*O*-rutinoside-5-*O*-glucoside, pelargonidin-3-*O*-sambubioside, pelargonidin-3-*O*-sambubioside-5-*O*-glucoside, pelargonidin-3-*O*-sophoroside-5-*O*-(malonyl)-glucoside, pelargonidin-3-sophoroside-5-glucoside. The fact that the number of authentic anthocyanin standards used here far exceeds the number of the total anthocyanins detected indicates that this analysis highly possibly detected all possible anthocyanins present in these samples.

Anthocyanin detection largely depends on the authentic anthocyanin standards used and no anthocyanins beyond the standards could be possibly detected. At the infant age of anthocyanin detection, there was often one or couple of anthocyanins identified due to the lack of authentic standards (Ashtakala & Maloney, 1971; Ng & Thimann, 1962). Afterwards, electrospray and tandem mass spectroscopy combined with HPLC was utilized to characterize anthocyanins and currently it is still the main methodology (Giusti, Rodríguez-Saona, Griffin, & Wrolstad, 1999). With the increasing number of identified authentic standards, more and more anthocyanins were detected (Benvenuti, Brighenti, & Pellati, 2018; Delgado-Povedano, de Villiers, Hann, & Causon, 2021; Kaiser, Müller-Ehl, Passon, & Schieber, 2020; Maldini et al., 2016; J. Wang, Kalt, & Sporns, 2000) Still, it is rather rare to find a report with more than 10 authentic standards used (Zhou & Yang, 2022). Here, 118 authentic anthocyanin standards are employed to identify these in the samples and this is so far the most exhaustive identification of anthocyanin with the most authentic standards used to the best of our knowledge.

## 4 Conclusions

With much more authentic anthocyanin standards and advanced MS/MS detection mode employed, a comprehensive analysis of the anthocyanin components in common anthocyanin-rich berries and grains in China is performed. Here, it is found that cyanidin-3-*O*-glucoside is the overwhelmingly major anthocyanin in all the berry and grain examined, which content in these samples varies a little and accounts for an average of 82% of the total anthocyanins. Peonidin-3-*O*-glucoside is the second most abundant anthocyanin in different samples, ranging from 2.6%–14.9% of the total anthocyanins. Pelargonidin-3,5-*O*-diglucoside is only found in blue honey suckle, and besides that, berries and grains share a dominant portion of common anthocyanins among them. This survey primes the establishment of a nutrition database of anthocyanin content in Chinese foods.

## CRediT authorship contribution statement

**Boyu Xie:** Visualization, Investigation. **Miaoshu Wang:** Investigation. **Dong Yang:** Writing – review & editing, Writing – original draft, Supervision, Project administration, Funding acquisition, Conceptualization.

## Declaration of competing interest

The authors declare that they have no known competing financial interests or personal relationships that could have appeared to influence the work reported in this paper.

## Data Availability

Data will be made available on request.
